# Anisotropic wood-hydrogel composites: Extending mechanical properties of wood towards soft materials’ applications

**DOI:** 10.1016/j.mtbio.2023.100772

**Published:** 2023-08-18

**Authors:** Sophie Marie Koch, Christian Goldhahn, Florence J. Müller, Wenqing Yan, Christine Pilz-Allen, Cécile M. Bidan, Beatrice Ciabattoni, Laura Stricker, Peter Fratzl, Tobias Keplinger, Ingo Burgert

**Affiliations:** a Wood Materials Science, Institute for Building Materials, ETH Zurich, 8093 Zurich, Switzerland; b WoodTec Group, Cellulose & Wood Materials, Empa, 8600 Duebendorf, Switzerland; c Soft Materials Group, Department of Materials, ETH Zurich, 8093 Zurich, Switzerland; d Department of Biomaterials, Max Planck Institute of Colloids and Interfaces, 14476 Potsdam, Germany

**Keywords:** Delignified wood, Composites, Hydrogel, Soft materials, Mechanical gradient, Cell alignment

## Abstract

Delignified wood (DW) offers a versatile platform for the manufacturing of composites, with material properties ranging from stiff to soft and flexible by preserving the preferential fiber directionality of natural wood through a structure-retaining production process. This study presents a facile method for fabricating anisotropic and mechanically tunable DW-hydrogel composites. These composites were produced by infiltrating delignified spruce wood with an aqueous gelatin solution followed by chemical crosslinking. The mechanical properties could be modulated across a broad strength and stiffness range (1.2–18.3 MPa and 170–1455 MPa, respectively) by varying the crosslinking time. The diffusion-led crosslinking further allowed to manufacture mechanically graded structures. The resulting uniaxial, tubular structure of the anisotropic DW-hydrogel composite enabled the alignment of murine fibroblasts *in vitro*, which could be utilized in future studies on potential applications in tissue engineering.

## Graphical abstract

**Figure:**
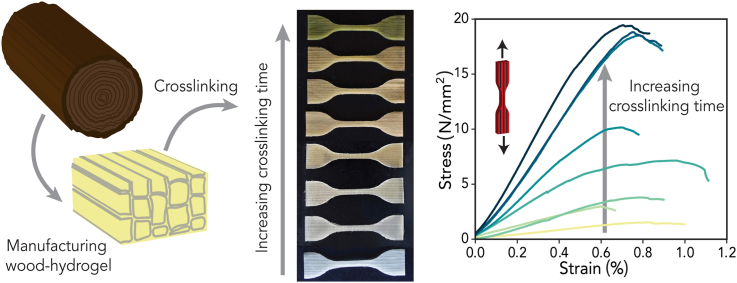

## 1 Introduction

Fiber-reinforced structures are prevalent in many biological materials, providing a multitude of properties due to their hierarchical, fibrous, and anisotropic arrangement at varying length scales. These biological materials are composed of a limited set of chemical constituents such as polysaccharides, proteins, and minerals, which jointly result in optimized mechanical properties and are able to fulfill various biological functions [1]. For example, wood and soft biological tissues, such as tendons or ligaments, both exhibit hierarchical, fibrous, and highly anisotropic structures (Fig. 9 I-II). In wood, the lignified cellulosic fibers and vessels are optimized for water transport and mechanical stability of trees. In tendon tissue, stiff collagenous fibers are axially aligned to efficiently transfer muscle generated tensile forces to the body's bone structure and to provide joint stability [2,3]. Similar fibrous structures are observed in many biological materials despite varying operating conditions, indicating their crucial role for material organization and corresponding properties.

Consequently, these biological materials have been an inspiration for engineered materials in many research and application areas [4]. For example, in the field of tissue engineering, biomimetic scaffolding materials have become state-of-the-art as cells should ideally experience a comparable micro-environment to their natural tissue to promote effective reconstruction of functional tissues [3,5,6]. Besides inducing a positive biological response of the cells, these materials should provide the appropriate environment for the regeneration of the natural tissue. Over the past two decades, cellulose nanocrystals (CNC) and nanofibrils (CNF) have been used for biomimetic scaffolds as stiff reinforcing fillers for soft hydrogel matrices because they exhibit inherent biocompatibility and biodegradability [7]. Simultaneously, the fast advance of manufacturing techniques, such as electrospinning [8] or 3D-printing methods [9], enables the assembly of such nano-scale building blocks into structurally demanding scaffolds. Still, the preceding disassembly of cellulosic resources into CNC and CNFs requires harsh chemical and/or energy intensive mechanical treatments [7]. Although offering a high freedom of structural design, additive manufacturing techniques still face numerous challenges, such as a limited resolution by nozzle size, void formation during printing, and poor interlayer adhesion [10].

Instead of using these bottom-up methods, we employed a top-down approach to create a bio-composite by utilizing wood as a structural component, which naturally possesses anisotropic properties at various hierarchical levels. Our overarching goal was to create a soft wood-based composite with tunable mechanical properties that could be applied in the future in other biological contexts, such as the biomedical field. Native wood is a stiff material with slow biodegradation. The first step to transform it into a soft composite that could be applicable for biomedical applications is a structure-retaining delignification process. This process renders wood malleable in the wet state while preserving cellulose fibril alignment on a nano-scale as well as tissue and cell alignment on a micro-scale [11]. Delignified wood (DW) offers a versatile platform for composite materials by facile combination with different matrices, resulting in very stiff and strong (thermosetting matrices) or soft and flexible (hydrogel and elastomeric matrices) composites [[12], [13], [14], [15]]. Recent research has demonstrated the potential of DW-hydrogel composites for tissue engineering and regenerative medicine approaches, e.g., for bone repair [[16], [17], [18]] or cell recruitment [19,20], and as an intervertebral disc substitute [21]. However, the mechanical properties of these composites are dictated solely by the mechanical properties of their constituents (reinforcing fiber and matrix), which limits their modifiability. By using a crosslinkable hydrogel matrix, the mechanical properties can be additionally modulated by the degree of crosslinking.

**Fig. 1:**
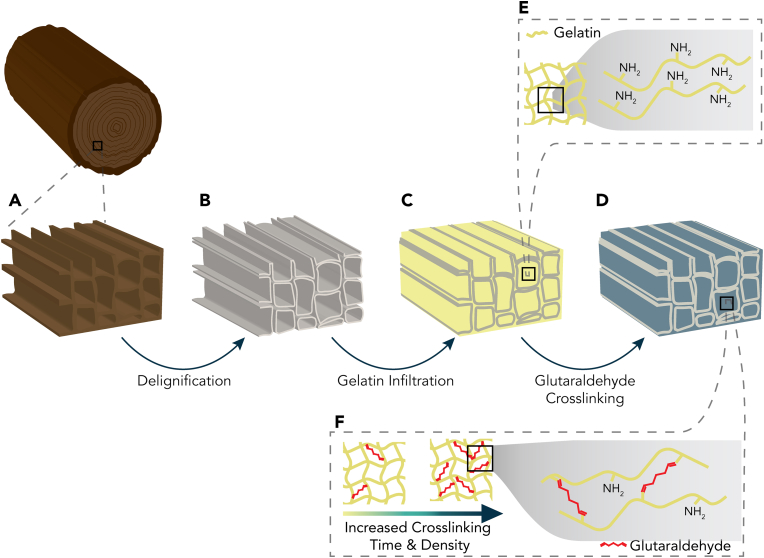
Schematic of crosslinked delignified wood-gelatin composite (DW-Gel) manufacturing process. (A) Native wood is (B) delignified and (C) infiltrated with gelatin and (D) further crosslinked in an aqueous glutaraldehyde solution. (E) Gelatin protein chains with lysine residues and free amino groups within the DW structure. (F) The crosslinking degree of gelatin protein chains can be tuned by adjusting the crosslinking time in glutaraldehyde solution.

In this research, we present a method to fabricate anisotropic and mechanically tunable DW-hydrogel composites with the additional possibility of incorporating a mechanical gradient (Fig. 1). We infiltrated delignified spruce wood with an aqueous gelatin solution (DW-Gel) followed by chemical crosslinking. The crosslinking step allows for modulation of the composites' mechanical properties by variation of the crosslinking time. Preliminary biocompatibility *in vitro* tests demonstrated that DW-Gel substrates support high cell proliferation and that their uniaxial, tubular structure induced cell alignment.

## 2 Materials and methods

### 2.1 Sample preparation

Radial-cut 1.3 mm thick Norway spruce (*Picea abies*) veneers (100 × 100 mm^2^) with an average density of 0.41 g/cm^3^ (±0.02) were delignified in an equal-volume mixture of hydrogen peroxide (35 wt% in water, Acros Organics) and glacial acetic acid (Fisher Chemicals) [22]. After delignification, the samples were washed with deionized water until the washing solution reached a pH value of 7.

For impregnation, 20 wt% low gel strength bovine gelatin (VWR, bloom number 90–130) was dissolved at 50 °C in deionized water. Then, the water-soaked delignified veneers were immersed in the gelatinous solution for 48 h at 50 °C under constant stirring. At the end of the impregnation cycle, the gelatin bath containing the samples was subjected to 50 mbar vacuum to remove residual entrapped air. Lastly, the surface of the impregnated samples was washed with cold deionized water to remove excess gelatin.

Crosslinking was performed in a glutaraldehyde (Fisher Chemical) solution (1 vol% in tris(hydroxymethyl)aminomethane (Biosolve) buffer, 100 mM, pH 8.5). The DW-Gel samples were immersed in the solution for various times (5, 10, and 30 min, 2, 4, 8, and 24 h) and subsequently washed three times in deionized water to remove unreacted glutaraldehyde.

For crosslinking gradients, DW-Gel samples were cut into 3 cm wide stripes (30 × 100 mm^2^, longitudinal x radial) and partially immersed into a beaker containing 100 mL of crosslinking solution. Thereby, the samples were immersed in the solution for approximately 40 mm of their radial direction for a diffusion-led crosslinking reaction. The set-up was covered with a 3 L beaker to avoid evaporation and air-drying of the samples and left for 16 h. Afterwards, the samples were washed with deionized water to remove unreacted glutaraldehyde.

To test the pure non-crosslinked and crosslinked gelatin matrix, a 20 wt% gelatin solution in deionized water was prepared at 50 °C under constant stirring. 15 mL of the solution was poured into a poly (ethylene) petri dish with a diameter of 85 mm to obtain a 2.1 mm thick gelatin film. The gelatin was cooled in the fridge for 24 h and subsequently crosslinked in the above mentioned glutaraldehyde solution for the same amount of time as the DW-Gel samples.

### 2.2 Sample characterization

#### 2.2.1 Microscopy

For fluorescence microscopy, the water-soaked (crosslinked) DW-Gel samples were frozen in liquid nitrogen and subsequently cut with a microtome (Leica RM2255) in the frozen state to obtain polished surfaces. Fluorescence microscopy was performed on a confocal microscope Leica SP8 with a 440 nm laser and on a Zeiss Axio Zoom V16 stereomicroscope using the Zeiss filter sets 49 (Excitation 365 nm, Emission 445 nm, blue), 38 (Excitation 470 nm, Emission 525 nm, green) and 20 (Excitation 546 nm, Emission 575 nm, orange).

For scanning electron microscopy, the samples were either cut by ultramicrotomy (UC7, Leica) for native and delignified wood or by using a razor blade on liquid nitrogen-frozen (DW-Gel samples). Then, the DW and DW-Gel samples (shown in Fig. SI 8) were freeze-dried using an Alpha 1-2LDplus (Christ) for 24 h at 0.12 mbar and 48 h at 0.05 mbar. All samples were glued onto SEM stubs, and edges were coated with silver paint. An 8 nm thick Pt/Pd sputter coating was applied using a Safematic CCU-010. Scanning electron microscopy (SEM) was conducted on a Hitachi SU5000 microscope using 5 kV acceleration voltage and a secondary electron detector.

#### 2.2.2 Gelatin content in DW-Gel

For the determination of the gelatin content in DW-Gel, approximately 1.2 g of fully dried DW-Gel was cut into small strips and immersed in 15 mL of deionized water. The slurry was then heated to 90 °C in a round-bottom flask using a water bath, while constantly stirring for 15 min. Subsequently, the slurry was filtered using a paper filter (MN 615) and all remaining fibers were rinsed out of the flask into a petri dish. The wet DW fibers were dried in an oven at 103 °C for 72 h and subsequently weighed. The remaining mass relates to the DW content in DW-Gel, while the gelatin content (Gel_content_) (%) was calculated using.

Equation 1

(1) $${Gel}_{content}=\frac{m_{DW-Gel}-m_{DW\;fiber}}{m_{DW\;fiber}}$$

with m_DW-Gel_ = mass of the DW-Gel samples in dry state and m_DW fiber_ = mass of the DW fibers after dissolving and rinsing off the gelatin.

#### 2.2.3 Water desorption and absorption

After crosslinking, the samples were weighed in the water-soaked state. Then, the samples were dried at 40 °C until reaching mass consistency and subsequently immersed in deionized water for 1, 5, 10, 30 min, 1, 2, 4, 8 h and weighed at each water absorption stage. Water desorption and absorption were calculated using.

Equation 2

(2) $$W_{ab,des}=\frac{m_{w}-m_{d}}{m_{d}}$$

with W_a__b__,des_ = water absorption or desorption (%), m_w_ = mass of the sample in the wet state and m_d_ = mass of the sample in the dry state.

#### 2.2.4 Ninhydrin staining and UV–Vis spectroscopy

The extent of amino group depletion of gelatin, caused by crosslinking with glutaraldehyde, was determined by a ninhydrin (2,2-dihydroxy-1,3-indanedione, Sigma-Aldrich) assay according to Yao et al. [23] In short, a 2 wt% solution in acetone was freshly prepared and 0.5 g of (crosslinked) DW-Gel samples were cut and crushed into small pieces. 10 mL of ninhydrin solution was pipetted into a round-bottom flask containing the sample. The solution was heated to 65 °C for 3 min in a water bath. Then, the solution was filtered through a 0.45 μm PTFE-filter using a 5 mL syringe. The solution was filled into Quartz cuvettes and light absorbance was measured using a Perkin Elmer Lambda 605 UV-Vvis spectrophotometer. The crosslinking degree is determined by the following equation:

Equation 3

(3) $$Crosslinking\;degree=\frac{{abs}_{DW-Gel}-{abs}_{CL}}{{abs}_{DW-Gel}}$$

with abs_DW-Gel_ = absorbance of DW-Gel at 581 nm and abs_CL_ = absorbance of different crosslinking times of DW-Gel samples at 581 nm.

#### 2.2.5 Rheology

The samples were air-dried and rehydrated prior to testing. The (non-)crosslinked DW-Gel samples were then cut into circles with a diameter of 20 mm using a hole punch. For the oscillatory rheological analysis, an Anton Paar MCR502 rheometer equipped with two rough parallel plates (diameter: 20 mm, 100 μm pillars) was used. A water-soaked sponge was placed around the sample to avoid air-drying during testing and a normal force of 5 N was applied to ensure uniform contact with the sample surface. For strain sweep experiments, a fixed frequency of 10 rad/s and temperature of 25 °C were applied and the strain was increased stepwise from 1E-5 to 1%. For temperature sweep experiments, the frequency and strain were fixed to 10 rad/s and 1E-4%, respectively, while the temperature was increased from 10 °C to 50 °C at a rate of 2 °C/min. For cyclic compression tests, an Ares G2 rheometer was used with an immersion cell. The samples were cut circularly to a diameter of 25 mm, and kept immersed in water during the measurement. The temperature was kept constant at 37 °C and the strain was cycled ±1% at a frequency of 1 s^−1^ for 24 h, with a preload of 5 N.

#### 2.2.6 Tensile testing

For macro-tensile tests, the specimens were cut into dog-bone shapes using a punching mold (Fig. 5A). Prior to testing, the tensile samples were air-dried and rehydrated to ensure a constant water content of all samples. Tensile tests were performed at a constant displacement rate of 1 mm/min on a universal testing machine ZwickRoell Z100 equipped with a 1 kN load cell.

**Fig. 2:**
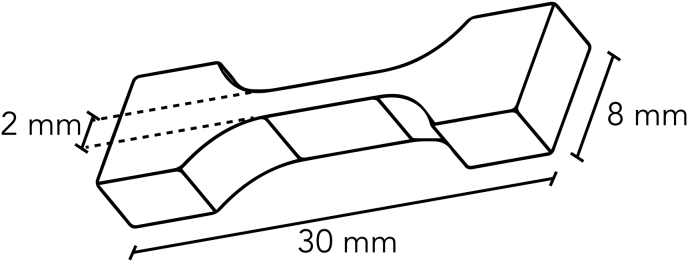
Geometry of the tensile samples used for micro-tensile tests.

For micro-tensile testing, the graded crosslinked samples were frozen at −18 °C for 2 h and dog-bone-shaped specimens were cut out of earlywood sections using a punching mold as shown in Fig. 2. To test the influence of the crosslinking gradient on the mechanical properties, three regions were defined, ranging from A (non-crosslinked), B (intermediate crosslinked as a result of crosslinking solution diffusion) to C (highly crosslinked as a result of immersion in the crosslinking solution) (Fig. 8). The specimens were air-dried and immersed into deionized water. The wet specimens were mounted onto a micro-tensile apparatus with a span length corresponding to the tapered part of the specimens. Fine grit sandpaper (600) was attached to the metal clamps using a fast-drying glue (UHU, Sekundenkleber) to avoid slippage. The micro-tensile apparatus consists of a linear table driven by a step motor and a 50 N load cell, similar to the setup described by Burgert et al. [24] The displacement was measured by means of video extensometry using two-line markers at the sample's mounting clamps. The testing speed was set to 10 μm/s.

Additionally, the pure (crosslinked) gelatin films were cut as described above. After drying, the samples were rehydrated in a 2:3 solution of deionized water:ethanol for 24 h according to Bigi et al. [25] to ensure a similar moisture content of all samples. The wet samples were tested on the same micro-tensile stage as described above but equipped with a 5 N load cell and the testing speed was set to 50 μm/s.

#### 2.2.7 Atomic force microscopy

Cross-sections of crosslinked DW-Gel samples for atomic force microscopy (AFM) were polished with an ultramicrotome (U7C8, Leica) equipped with a diamond knife (Diatome) in the dry state. Prior to AFM mapping in water, the samples were immersed in Milli-Q water for a minimum of 72 h. Quantitative Imaging (QI™) was performed on a JPK Bruker Nanowizard 4 with a pyramid-shaped tip (CONTR-10, Nanoworld) on a cantilever with a nominal force constant of 0.2 N/m and resonance frequency of 13 kHz. The cantilever was calibrated using a contact-based method by performing three force-distance (F-D) measurements on a glass slide immersed in water. The sensitivity and the force constant are reported as the arithmetic mean (± standard deviation) in Table SI 3. The QI-mapping was performed with a constant speed and setpoint and the retraction was set to 1000 nm. For analysis of the F-D curves, the JPK image processing software (JPK Instruments AG - Bruker Nano GmbH, Germany) was used.

#### 2.2.8 Cell morphology and proliferation

Crosslinked DW-Gel samples (30 min and 24 h) were cut into 12-mm diameter circular samples using a hole puncher, and their surface was prepared with a microtome blade by hand. Before cell seeding, all samples were sterilized in 70 vol% ethanol solution for 1 h and washed twice in PBS solution. Additionally, the samples were sterilized by UV-light exposure for 1 h per substrate side.

Murine NIH3T3 fibroblasts were incubated in culture medium (Dulbecco's Modified Eagle's Medium-High Glucose (Sigma), 10% FBS superior (Sigma), 10 μg/mL gentamicin (Sigma)) and passaged four times before cell seeding. For cell seeding, 50 μL of cell suspension containing 5 x 10^4^ cells/cm^2^ in culture medium was pipetted onto the sample surface. After 1 h, the remaining 1450 μL culture medium was carefully added to the petri dish. The samples were then incubated in a humidified atmosphere with CO_2_ (5%) at 37 °C for 1, 3, and 7 days. Three samples per condition were stained after different incubation times with Hoechst 33,342 (Invitrogen R37605) and Calcein AM/PI (Invitrogen R37601) staining.

The mitochondrial activity was determined using an EZ4U cell proliferation and cytotoxicity assay (Biomedica). For this, 9 samples of each variant (30 min and 24 h) were seeded with cells as described above. After an incubation time of 1, 3, and 7 days, the EZ4*U* test was performed on three samples per variant. The culture medium was removed before adding activator-substrate mixture. After incubation for 3 h at 37 °C, 3 x 200 μL suspension were pipetted onto a 96 well-plate and absorbance was measured at 450 and 630 nm (cell residues) with a microplate-reader (Agilent Technologies) according to the EZ4U protocol. The average OD 450 after one day incubation of three wells containing only seeded cells but without sample, was taken as a reference to describe 100% viability. Therefore, the cell proliferation (%) is the ratio of the absorbance of the sample-containing wells to the reference.

#### 2.2.9 Fiber and cell orientation image analysis

The orientation of wood fibers and cultivated cells were analyzed using the stereomicroscopy images collected with the filter sets 20 (Excitation 546 nm, Emission 575 nm, orange) and 38 (Excitation 470 nm, Emission 525 nm, green). In the orange channel, only the wood fibers are visible. To evaluate their directionality, we determined the orientation of the local gradient of the image intensity function at each pixel by applying a 5 x 5 Sobel filter [26]. The implementation was performed by means of the ImageJ plugin ‘Directionality’. For evaluation of the cells' directionality, the full profiles of the cultivated cells were extracted and their shapes were analyzed. To this aim, the green images were pre-processed to remove the background. First, the orange channel, where only the fibers are visible, was subtracted from the green channel. Subsequently, we enhanced the brightness contrast, applied a smoothing filter that replaced each pixel with the average value of the surrounding 3 x 3 square, and applied a “rolling ball” background subtraction [27]. We binarized the resulting image by thresholding it, thus obtaining a black and white image, where the cells appeared as white areas. The cells were eventually detected, and their shape was analyzed. In particular, the angle between the main longitudinal axis of the fitting ellipse and the horizontal border of the frame was used as an indicator of the cell's direction. The described procedure was performed in ImageJ [28], by means of the native Fiji plugins Brightness/Contrast Adjustment, Smooth, Subtract Background, and the Biovoxxel ‘Extended Particle Analyzer’ plugin, in combination with an in-house code written in Matlab.

## 3 Results and discussion

### 3.1 Wood structure-retaining composite manufacturing

**Fig. 3:**
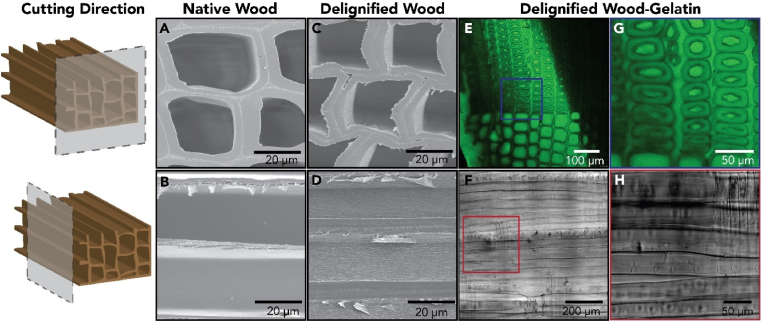
Microscopic images of native wood, delignified wood, and DW-Gel. Scanning electron microscopy images of (A, B) the cross-section and longitudinal-tangential surface of native spruce and (C, D) the cross-section and longitudinal-tangential surface of delignified spruce. (E, G) Confocal fluorescence microscopy images of DW-Gel's cross-section. (F, H) Phase contrast images of the longitudinal-tangential surface of DW-Gel.

For the preparation of DW-Gel bio-composites, spruce wood veneers were delignified using a hydrogen peroxide/acetic acid solution [11,22]. Native wood tissue comprises hollow cells (fibers) with a high aspect ratio, which are aligned in the longitudinal direction of the tree's stem axis (Fig. 3A and B). Fig. 3C and D shows the preserved natural wood tissue structure after the delignification treatment. While cell walls appear slightly sheared due to microtome-cutting, the delignified fibers remain aligned, and the wood cell lumina remain accessible. In the second step, the delignified wood samples were infiltrated with an aqueous gelatin solution. As gelatin emits an autofluorescence response in the green wavelength (Fig. SI 1), confocal fluorescence microscopy images of DW-Gel composites allow for confirmation of the effective hydrogel matrix impregnation (Fig. 3E, G). They show the homogeneous infiltration of the cell lumina, the intercellular areas, and the cell walls in the cross-sections. This is in contrast to pure DW with only poorly visible tissue structure due to low autofluorescence response (Fig. SI 2). On average, the DW-Gel composites contain 36.5% of dry gelatin (±2.2%; Tab. SI 1).

The upper critical solution temperature (UCST) of gelatin (ca. 35 °C) leads to the dissolution and disassembly of the composite at elevated temperatures. Chemical crosslinking is a well-known method to stabilize gelatin hydrogel networks for temperatures above the UCST [29]. Here, gelatin was crosslinked by glutaraldehyde at high pH, which is governed by Schiff base formation [[30], [31], [32]].

### 3.2 Mechanical modulation and gradient structures by variation of the crosslinking degree

**Fig. 4:**
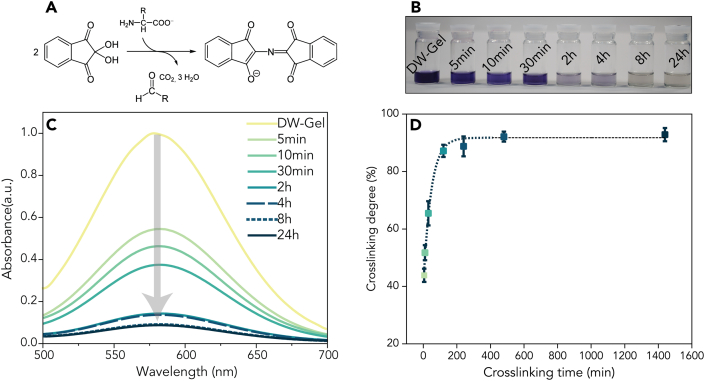
Ninhydrin assay showing the gradual decrease of available amino groups with increasing crosslinking time. (A) Reaction of ninhydrin with primary amine groups of, e.g., gelatin, resulting in a so-called Ruhemann's purple. (B) Photograph of the ninhydrin solutions displaying a color intensity decrease with increasing crosslinking time. (C) UV–Vis absorbance of (crosslinked) DW-Gel in the range of 500–700 nm. (D) Depletion of amine groups determined by relative UV–Vis absorbance at peak 581 nm determined by Equation (3). (For interpretation of the references to color in this figure legend, the reader is referred to the Web version of this article.)

We varied the crosslinking time of the DW-Gel samples, profiting from the facile two-step (infiltration and crosslinking) manufacturing process. We crosslinked DW-Gel for 5 min, 10 min, 30 min, 2 h, 4 h, 8 h, and 24 h and investigated the corresponding crosslinking degree by ninhydrin staining (Fig. 4). Staining with ninhydrin is a commonly used method for monitoring the depletion of free amino groups during crosslinking reactions of lysin-containing proteins, such as gelatin (Fig. 4A) [23,33,34]. At a pH between 7 and 9, glutaraldehyde reacts with the amino groups of gelatin with minor reversibility [35]. Whereas the crosslinking reaction initially takes place rapidly, the reaction rate slows down successively [35]. Unreacted amino groups react with ninhydrin to form a purple color, the so-called Ruhemann's purple, which can be quantified using a UV–Vis spectrophotometer. Consequently, a high number of unreacted amino groups correspond to a deep purple color, while samples with many depleted (crosslinked) amino groups show a brighter, less purple color.

**Fig. 5:**
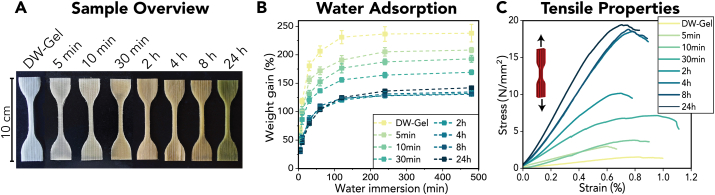
Physical characterization of DW-Gel composites crosslinked for different time durations. (A) Photograph of macro-scale tensile samples. (B) Weight gain by water absorption over 8 h (n = 3). (C) Representative example of tensile curves.

Here, we observe a visible decrease in color intensity of DW-Gel samples up to 8 h crosslinking time (Fig. 4B). Spectrophotometry confirms the visual impression of a decreasing absorbance at a wavelength of 581 nm from non-crosslinked samples up to 24 h crosslinking time (Fig. 4C). Fig. 4D shows the crosslinking degree of the DW-Gel as calculated with Equation (3) as a function of the crosslinking time. Already after 5 min, a crosslinking degree of over 40% is achieved and the reaction proceeds very rapidly up to 2 h. Further, the crosslinking degree approached a maximum asymptote up to 90%. Since the reaction of amino groups with ninhydrin to Ruhemann's purple is a multi-step reaction via the formation of imine bonds, these crosslinking degree values should not be considered absolute values but rather as an indication of the evolution of the crosslinking degree over time.

The crosslinking degree determines the physical and mechanical properties of hydrogels [25]. Fig. 5A shows the crosslinked DW-Gel tensile test samples. The color of the DW-Gel samples changes from white to yellow to green with increasing crosslinking time. Crosslinking of DW-Gel samples for longer durations results in decelerated water absorption and a lower maximum amount of absorbed water (Fig. 5B). Similar trends have been observed with alternative crosslinkers, such as genipin [36]. The decelerated water absorption results in reduced viscoelastic restoring forces and lower swelling degrees [13,36,37]. For crosslinking times higher than 2 h, we observe no further decrease in water uptake, which reasserts the high crosslinking degree found in Fig. 4D at that time point. Fig. 5C and Tab. SI 2 show the macro-tensile properties of crosslinked DW-Gel in the fiber direction. There is a clear trend of increasing stiffness and strength with longer crosslinking times. The composites reach a maximum value of around 18 MPa tensile strength and ∼1300 MPa tensile stiffness at a crosslinking time of 4 h. In general, the mechanical properties of DW-Gel samples cover a broad range of strength and stiffness values (1.2–18.3 MPa and 170–1455 MPa, respectively). They also exceeded the mechanical properties of water-saturated pure delignified wood with a tensile strength of 0.12 MPa [38] and those of pure (crosslinked) gelatin samples by three or four orders of magnitude (Fig. SI 3).

During delignification the lignin-rich compound middle lamella, connecting single wood fibers and transferring stresses between them, is removed [39]. Aqueous gelatin impregnation fills these intercellular regions and connects the wood fibers via an artificial “gelatin middle lamella”, similar to a resin matrix in unidirectional fiber-reinforced composites. The mechanical properties of pure (crosslinked) gelatin therefore correspond closely to those of DW-Gel composites perpendicular to the fiber direction, where the fiber-embedding matrix (crosslinked gelatin) predominantly dictates the material behavior during tensile testing. Whereas non-crosslinked gelatin samples could not be tested due to their extreme fragility and swelling, crosslinked gelatin showed a substantial stiffness increase up to 30 min crosslinking duration. Due to failure in proximity to the clamping area of some samples, pure crosslinked gelatin samples could exhibit a slightly higher actual mean strength than the ultimate stresses shown in Fig. SI 3. For longer crosslinking times, there is a slight decrease in mechanical properties. Similarly, Liu et al. observed that the tensile strength and elastic modulus of isotropic chitosan/gelatin scaffolds increased to a certain degree of glutaraldehyde concentration, but decreased at concentrations higher than 8% [40]. The authors explained this trend with possible molecular degradation mechanisms of the chitosan/gelatin scaffolds caused by glutaraldehyde. This degradation could also negatively influence the stiffness of crosslinked pure gelatin at higher crosslinking times.

**Fig. 6:**
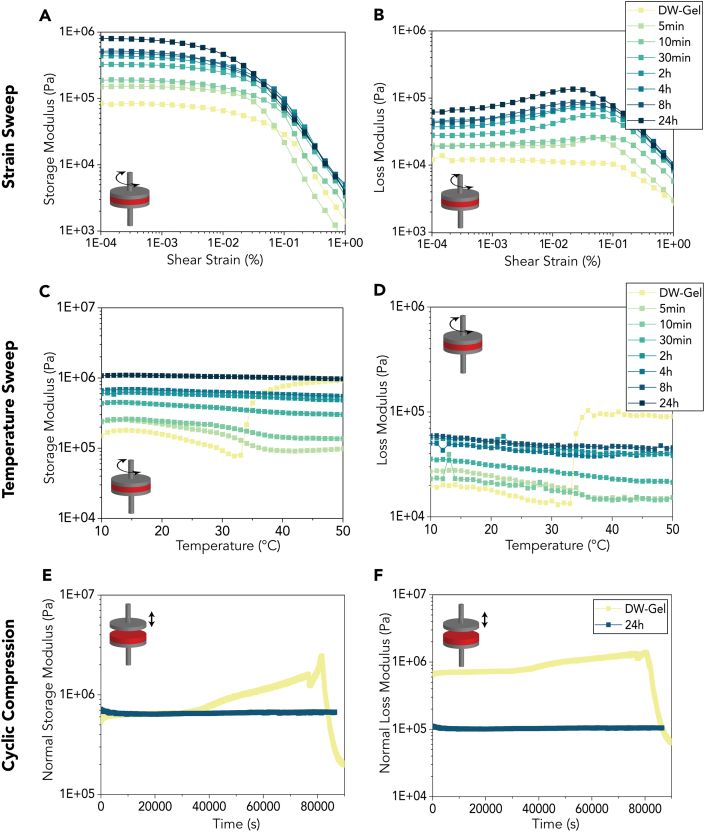
Rheological analysis of crosslinked DW-Gel. (A, B) Storage and loss modulus versus shear strain. (C, D) Storage and loss modulus in dependence of temperature. (E, F) Storage and loss modulus of non-crosslinked DW-Gel and DW-Gel crosslinked for 24 h tested at 37 °C for 24 h in cyclic axial compression tests.

Rheological strain sweep experiments show relatively high shear moduli, which can be traced back to the highly anisotropic tubular array structure of DW-Gel, showing a higher torsion resistance than disordered structures (Fig. 6) [41]. Moreover, the crosslinking degree of the DW-Gel samples is proportional to the magnitude of the storage (G′) and loss moduli (G″) (Fig. 6A and B). DW-Gel shear moduli are in the range of 1E5 to 1E6 Pa; hence, they fall within the viscoelastic range of dense connective tissues [33]. Prior to the final steep decline of G′ and G″ associated with structural instability of the composite at higher strains [29], the data showed an increase in loss modulus in the range of 1E-2% to 1E-1% shear strain. This increase reveals the possibility of dissipating energy, which is particularly interesting for higher-impact loadings.

Temperature sweep experiments reveal that all crosslinked DW-Gel samples are stable over a temperature ranging from 10 to 50 °C (Fig. 6C, D). For lower crosslinking durations, there is a small loss in storage moduli with increasing temperature, while DW-Gel crosslinked for over 30 min shows a predominantly linear behavior. For non-crosslinked DW-Gel, however, the composites lose structural integrity at temperatures higher than 34 °C, the onset temperature of gelatin dissolution. As the rheometer applied a normal force to equalize the slightly uneven composite surface structure, the distance between the plates decreases at higher temperatures, and consequently, we observe a steep increase in storage and loss moduli after the initial decrease at 34 °C (Fig. SI 4). Accordingly, cyclic compression tests at 37 °C (corresponding to the core body temperature) over 24 h show that non-crosslinked DW-Gel is unstable over a longer time (Fig. 6 E, F). Therefore, a certain crosslinking degree is necessary to make the DW-Gel stable at core body temperature. However, the required crosslinking degree might also depend on the desired biodegradation rate.

**Fig. 7:**
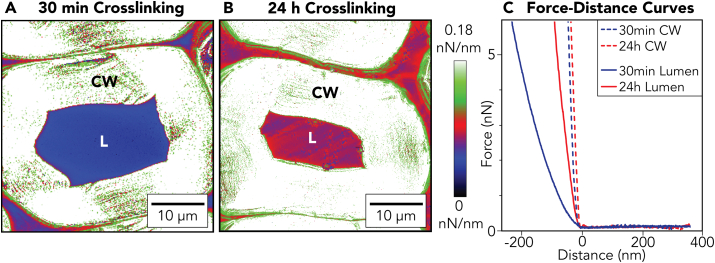
AFM force mappings of single DW-Gel wood cells crosslinked for (A) 30 min and (B) 24 h. (C) Shows force-distance curves of 30 min and 24 h crosslinked samples in the cell wall and lumina regions. Whereas the cell walls of the differently crosslinked samples are comparably stiff, the lumen filling of the 30 min crosslinked sample is softer than the 24 h crosslinked sample. Each curve was collected at a speed of 2.5 μm s^−1^ using a cantilever with a normal spring constant of 0.27 N/m. CW = Cell Wall, L = Lumen.

Fig. 7 shows AFM force mappings of single wood cells of DW-Gel crosslinked for 30 min (A) and 24 h (B), and representative force-distance curves of the cell wall and the gelatin-filled lumina regions (C). Additional force-distance curves can be found in Fig. SI 5. Within the cell wall, the stiffnesses of 30 min and 24 h crosslinked samples are rather similar. It is worth noting that cellulose fibrils are five to six orders of magnitude stiffer than hydrated gelatin [42,43]. Therefore, the altered stiffness of the gelatin by crosslinking plays only a minor role within the cell wall. However, in the gelatin-filled lumina, we observe an apparent increase in hydrogel stiffness with increasing crosslinking time. Compared to densely crosslinked DW-Gel (24 h), loosely crosslinked DW-Gel (30 min) contributes more repulsive interaction, which may result from strong swelling of loosely crosslinked chains that extend far into the water. Soft hydrogels such as gelatin are commonly tested with spherical AFM tips rather than triangular tips to avoid excessive indentation [44]. As we aimed for a maximal resolution to unravel the gelatin-filled wood cell structure, we used a triangular tip, and kept the same tip for comparability between the two force mappings (Tab. SI 3). Therefore, this data should serve as a basis for comparing the crosslinking durations but does not provide absolute values. According to the minimum of the retraction curves, the adhesion between AFM tip and gelatin-filled lumen surface should be negligible, revealing the effective hydration of DW-Gel in water.

**Fig. 8:**
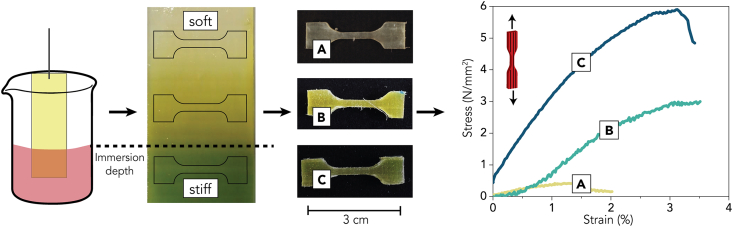
Manufacturing and testing of the gradient crosslinked DW-Gel. The crosslinking occurs by a diffusion-led reaction by immersing DW-Gel into a glutaraldehyde solution. Micro-tensile specimens are punched out of differently crosslinked DW-Gel regions and tested in micro-tensile tests.

**Fig. 9:**
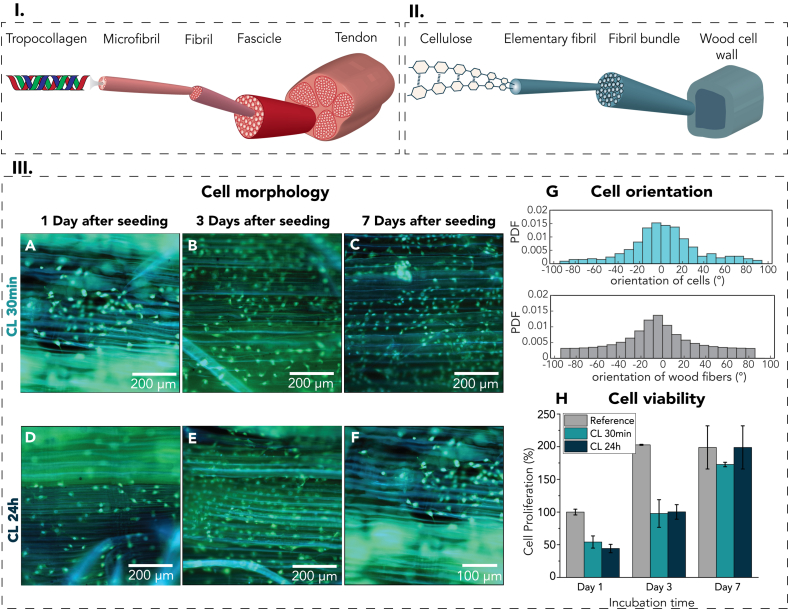
(I) The hierarchical, fibrous structure of a tendon consists of tropocollagen fibers. (II) The wood cell wall (S2-layer) exhibits a hierarchical, fibrous structure similar to the tendon. (III, A-F) Fluorescence stereomicroscopy images of NIH3T3 cells on DW-Gel 30 min and 24 h (III, G) Orientation of wood fibers of DW-Gel 30 min (grey) and orientation of cells (blue) cultured on the substrate after 7 days expressed as probabilty distribution function (PDF) vs. orientation angle. (III, H) Proliferation of cells cultured in DW-Gel containing wells. (For interpretation of the references to color in this figure legend, the reader is referred to the Web version of this article.)

The mechanical and rheological characterization of DW-Gel show the possible modulation of mechanical properties by crosslinking degree at several length scales. Tailorable mechanical properties over a wide strength and stiffness range could play a major role when designing a suitable biomimetic material for potential tissue engineering applications [45].

As an additional method, we can apply the crosslinking time-dependent mechanical modulation to manufacture DW-Gel with a mechanical gradient. As shown in Fig. 8, we immersed the composite into the crosslinking solution and obtained a color gradient exceeding the immersion depth. Overnight the crosslinking solution diffuses into the DW-Gel sample, inducing a yellowish crosslinked region reaching from the immersion border up to ∼2.5 cm above (region B). As known from previous tests, these color changes also indicate alterations in mechanical properties. We punched micro-tensile samples out of the differently crosslinked earlywood regions A (non-crosslinked), B (crosslinked by diffusion of crosslinking solution above immersion limit), and C (crosslinked in solution for 16 h). Micro-tensile testing shows the gradually increasing tensile stiffnesses and strengths (Fig. 8, Table 1, and Fig. SI 6) from region A to region C. Generally, the micro-mechanical properties are lower than the macro-mechanical properties, which can be attributed to scaling effects and the testing of pure earlywood regions with lower fiber content.

**Table 1:** Strength and E-modulus of the different regions (A, B, C) in gradient crosslinked DW-Gel with arithmetic mean ± standard deviation.

Gradient Region	Strength, mean (MPa)	Stiffness, mean (MPa)
A	0.41 ± 0.11	33.0 ± 10.7
B	2.62 ± 0.99	118.3 ± 35.4
C	5.25 ± 0.86	216.6 ± 56.5

In body tissues, material gradients help minimizing stress concentrations on junction sites. Particularly, tendon interfaces to muscles (myotendinous junctions) or bones (entheses) are optimized by structural and mechanical gradient structures to efficiently transfer the load to a wide area surrounding the junction [46]. Synthetically replicating these structures remains challenging despite enormous research efforts, often requiring highly specialized and expensive equipment [47]. The use of gradient structures to repair tendon-to-bone interfaces is a current research subject, aiming at functionally enhancing the integration of grafts. For instance, Chen et al. mineralized a nanofibrous scaffold in a graded manner, which was then used to wrap an autologous tendon graft [48]. This sheathed and grafted tendon showed enhanced integration in the tendon-to-bone interface after eight weeks. Here, we present a simple process to manufacture composites with mechanical gradients without stepped transitions, which could be similarly interesting for these complex interfacial areas. Moreover, Oh et al. found that stem cells differentiated into specific cell types, depending on the stiffness of the substrate [49]. Therefore, further experiments involving stem cell seeding on DW-Gel could be of interest to investigate cell differentiation in different stiffness ranges.

### 3.3 *In vitro* cell viability and alignment on DW-gel

The fibrous structure found in biological materials plays a crucial role for their mechanical properties and biological functions. Fig. 9 I and II highlight the structural similarities between tendon and wood, both exhibiting a hierarchical, axially aligned fibrillar arrangement. In biological tissues, collagen's fibrillar state is not only essential for the mechanical properties of the extracellular matrices but also for their interaction with cells.

To achieve *in vitro* structuration of tissues at the macroscopic scale, collagen-based scaffolds with aligned pores have been successfully manufactured using freeze-casting procedures [50]. These scaffolds exhibited pore diameters ranging from 7 to 19 μm and lengths from 50 to 270 μm, effectively guiding the alignment of fibroblasts and myoblasts *in vitro*. Similarly, spruce wood cells possess lumina diameters ranging from 6 to 45 μm, with even greater lengths of 1300–4800 μm [51].

Rather than relying on specialized equipment for anisotropic scaffold fabrication, we employed the DW-Gel composites to influence cell morphology and alignment. We seeded NIH3T3 murine cells onto the DW-Gel surface to analyze the effect of the wood fibers on fibroblast morphology. Since we used glutaraldehyde as a cost-effective model crosslinker, which is known for its cell toxicity [[52], [53], [54]], we additionally performed a glycine treatment on the composite before cell seeding to remove excess aldehyde groups [54,55]. In the following steps, we stained the cell-seeded DW-Gel after 1, 3, and 7 days with Hoechst 33,342 and Calcein AM/PI and analyzed the cell morphology and alignment by fluorescence stereomicroscopy (Fig. 9 III A-F). An increasing cell population with time and a preferential cell orientation were visible. Cell alignment was analyzed by fitting ellipses to the cell contours and determining their orientation (Fig. 9 III G, Fig. SI 7). A distinct alignment of cells in wood fiber direction could be measured, which indicated that the wooden cell structure could serve as contact guidance for living cells. Still, it is important to emphasize that cells were seeded on the surface of the composite. Proliferation and differentiation of cells can vary in 2D and 3D culture media [56]. To advance towards 3D cell culturing within DW-Gel composites, freeze-drying could be a feasible approach, for which we observed a highly porous double-network structure with two mesh sizes (Fig. SI 8).

Next to cell alignment and morphology, sufficient cell proliferation and density is essential for effective tissue regeneration. Fig. 9 III H shows the percentage of cell proliferation in reference to cell seeding after 0 h on a petri dish surface (= 100%). Initially, DW-Gel-containing wells show a significantly lower number of cells after day 1 and 3, but after day 7, the number of cells is comparable to that in the control. Consequently, DW-Gel does not emit critical toxic leachates and can be considered for further *in vitro* studies. Although these first results are promising in terms of cell proliferation and alignment, further experiments regarding cell adhesion, migration, and aggregation are required to evaluate the true potential of DW-Gel for tissue regeneration.

## 4 Conclusions

In this work, we developed a bio-composite using delignified spruce wood and gelatin. The composite can be tailored to exhibit a wide range of mechanical properties. Prolonged crosslinking times increase macro- and micro-mechanical properties of DW-Gels. Additionally, we implemented a mechanical gradient into the composite. The retained fibrous structure of wood in the composite facilitated the alignment of seeded fibroblasts through contact guidance. The mechanical adjustment of this aligned tissue structure could render DW-Gel a promising cell substrate for more in-depth *in vitro* and *in vivo* studies for tissue engineering applications requiring specific mechanical properties. Further research towards a potential application in tissue engineering needs to focus on investigating cell-substrate interactions, including cell adhesion, migration, and aggregation. Finally, stem cells could also be cultured on gradient DW-Gel substrates to test their potential to induce graded cell differentiation.

## Credit author statement

S.M.K.: Conceptualization, Methodology, Investigation, Data curation, Visualization, Writing; C.G.: Conceptualization, Methodology and Writing; F.J.M., W.Y., C.P-A., B.C., L.S.: Investigation, Data curation, Writing; C.M.B., P.F., and T.K.: Supervision, Writing; I.B.: Conceptualization, Supervision, Writing.

## Declaration of competing interest

The authors declare that they have no known competing financial interests or personal relationships that could have appeared to influence the work reported in this paper.

## Data Availability

Data will be made available on request.
