# Supplementary material for: Anisotropic wood-hydrogel composites: Extending mechanical properties of wood towards soft materials’ applications
Source: Mater Today Bio. 2023 Aug 18;22:100772. doi: 10.1016/j.mtbio.2023.100772 (PMC10477686; doi:10.1016/j.mtbio.2023.100772)
Supplement: Multimedia component 1 [file mmc1.pdf]

Supplementary Information

Anisotropic Wood-Hydrogel Composites: Extending Mechanical Properties of Wood Towards Soft Materials' Applications

Sophie Marie Koch[a,b*], Christian Goldhahn[a], Florence J. Müller[c], Wenqing Yan[a,b], Christine Pilz-Allen[d], Cécile M. Bidan[d], Beatrice Ciabattoni[a], Laura Stricker[a], Peter Fratzl[d], Tobias Keplinger[a], Ingo Burgert[a,b*]

[a] Wood Materials Science, Institute for Building Materials, ETH Zurich, 8093 Zurich, Switzerland.

[b] WoodTec Group, Cellulose & Wood Materials, Empa, 8600 Dubendorf, Switzerland.

[c] Soft Materials Group, Department of Materials, ETH Zurich, 8093 Zurich, Switzerland.

[d] Department of Biomaterials, Max Planck Institute of Colloids and Interfaces, 14476 Potsdam, Germany.

*Corresponding authors: Sophie Marie Koch (sokoch@ethz.ch) and Ingo Burgert (iburgert@ethz.ch)

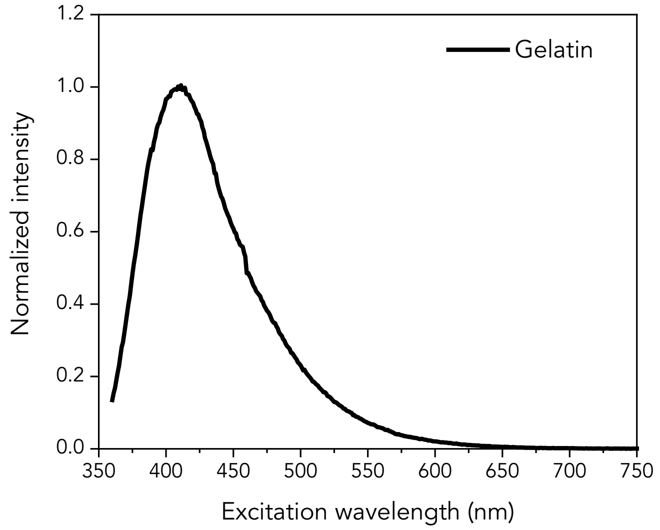

Figure SI 1. Fluorescence spectrum of 20wt% gelation solution at an excitation wavelength of 340 nm recorded with an Agilent Cary Eclipse spectrofluorometer. Although the maximum fluorescence response is at 411 nm, gelatin shows a fluorescence emission signal also at higher wavelengths in the green spectrum (490-575 nm).

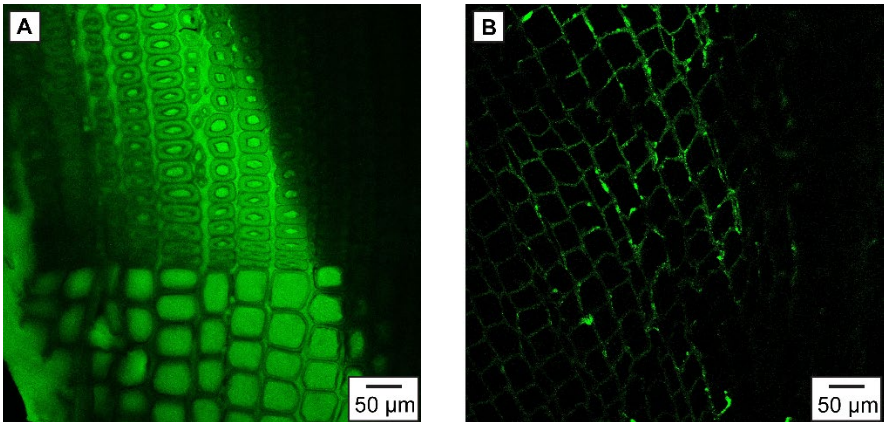

Figure SI 2. Fluorescence microscopy image of (A) DW-Gel and (B) delignified wood. Compared to DW-Gel, the pure delignified wood shows a lower fluorescent signal.

Table SI 1. Mean dry gelatin content in DW-Gel samples.

No.	DW-Gel, dry (g)	DW, dry (g)	Gelatin dry content (wt%)
1	0.2234	0.1466	34.4%
2	0.189	0.1205	36.2%
3	0.1923	0.1178	38.7%
Mean			36.5%
Std. dev.			2.2%

Table SI 2. Strength and E-modulus of non-crosslinked and crosslinked DW-Gel with arithmetic mean ± standard deviation.

	Strength, av. (MPa)	E-modulus, av. (MPa)
**DW-Gel**	1.2 ± 0.6	170 ± 81
**5min**	2.5 ± 0.4	115 ± 12
**10min**	3.7 ± 0.6	312 ± 66
**30min**	8.2 ± 1.5	689 ± 176
**2h**	13.1 ± 2.2	1075 ± 226
**4h**	18.3 ± 1.7	1298 ± 105
**8h**	18.2 ± 2.0	1293 ± 258
**24h**	17.1 ± 1.7	1455 ± 161

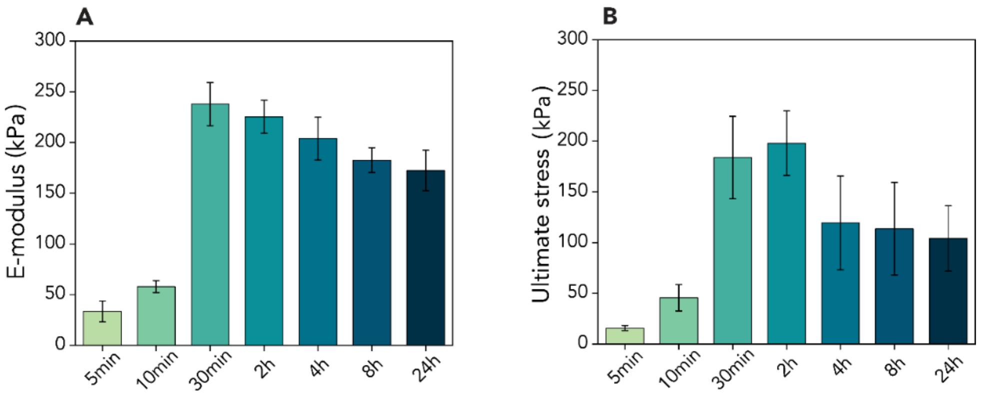

Figure SI 3. E-modulus (A) and ultimate tensile stress (B) of pure crosslinked gelatin tested in micro-tensile testing. Pure non-crosslinked gelatin could not be tested in the micro-tensile set-up due to instability in water.

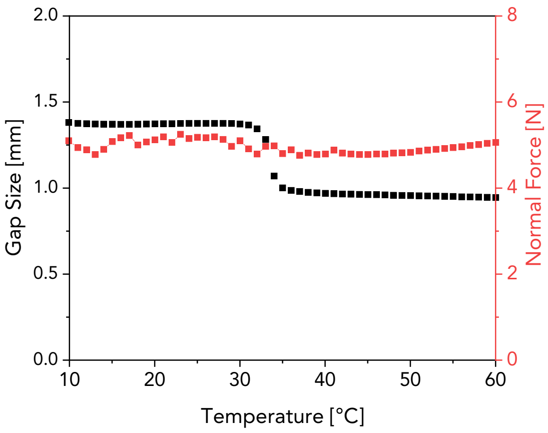

Figure SI 4. Gap size and normal force of non-crosslinked DW-Gel as a function of temperature corresponding to rheological data shown in Fig. 6 C and D. The normal force is controlled (5 N) during the experiment. At 34°C, the gap size decreases from ca. 1.3 mm to 0.9 mm as the non-crosslinked gelatin becomes mechanically instable.

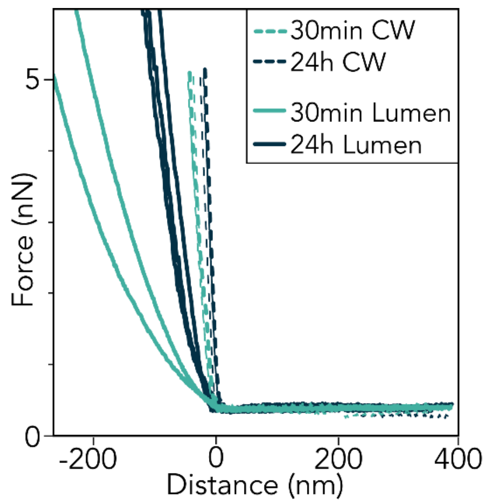

Figure SI 5. Representative AFM force-distance curves (n=3) of 30 min and 24 h crosslinked DW-Gel, gelatin-filled lumina and cell walls (CW), respectively.

Table SI 3. Sensitivity and spring constant of the used AFM tip on a stiff substrate (glass slide).

No.	Sensitivity (nm/V)	Spring Constant (N/m)
**1**	54.0	0.276
**2**	53.9	0.295
**3**	58.2	0.251
**Mean**	55.4	0.274
**Std. dev.**	2.5	0.022

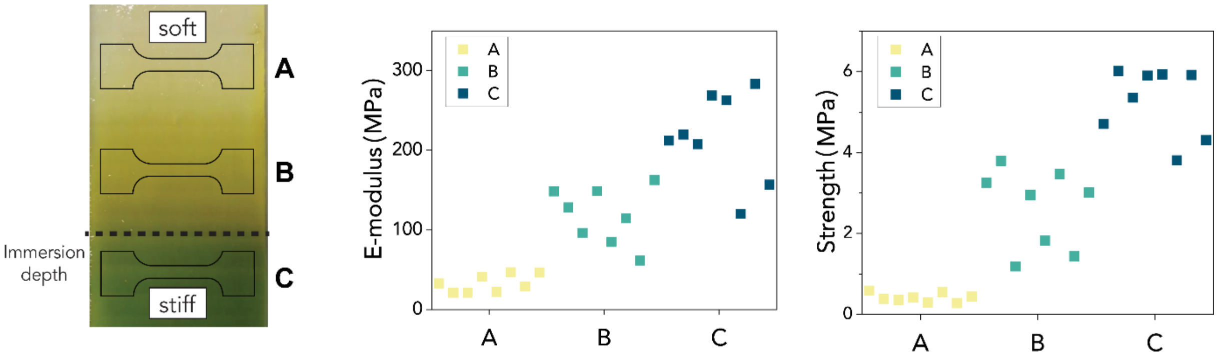

Figure SI 6. Micro-tensile testing of DW-Gel gradient in the regions A, B, and C. E-moduli and strength values of DW-Gel gradient samples.

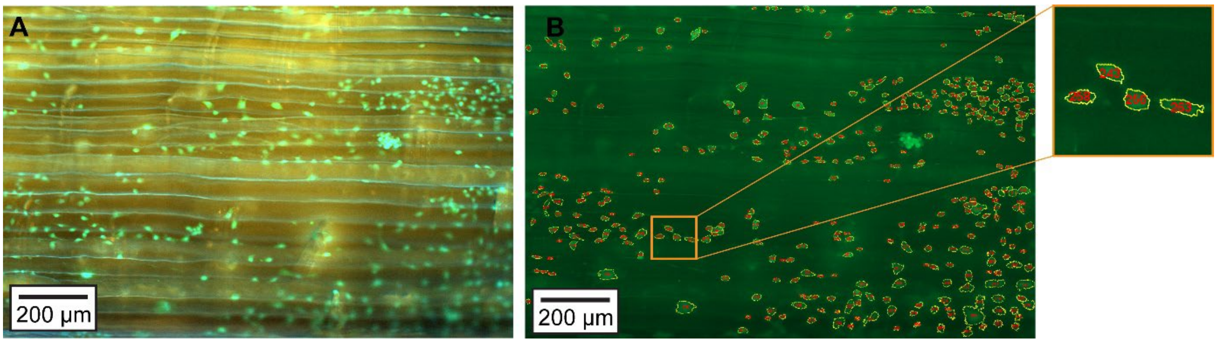

Figure SI 7. Fluorescence stereomicroscopy image of DW-Gel 30 min after 7 days. (A) Unprocessed microscopy image containing the orange, blue and green channel. (B) Processed image with fitted ellipses around cell contours for analysis of the cells' orientation. Cell numbers are written in red inside the cells as shown in the magnified image section.

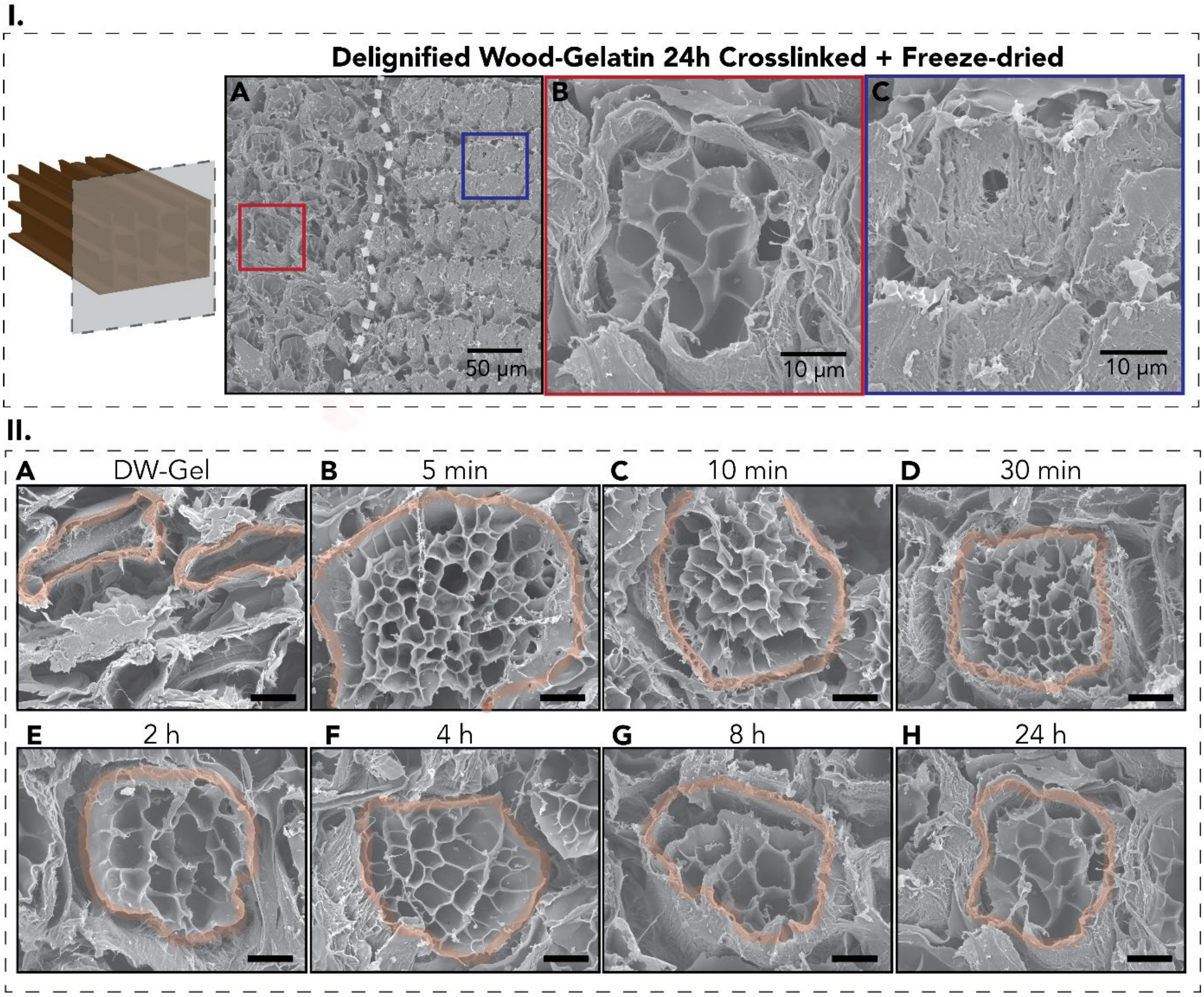

Figure SI 8. Scanning electron microscopy images of freeze-dried crosslinked gelatin-filled delignified wood. **I.A** shows an overview of crosslinked (24 h) gelatin-filled lumina of late- and earlywood. The annual growth ring in highlighted by the white dashed line. **I.B** and **C** provide a higher magnification of a single earlywood and latewood cell, respectively. **II.A-H** show an earlywood cell of non-crosslinked, 5 min, 10 min, 30 min, 2 h, 4 h, 8 h, and 24 h crosslinked samples, respectively. The inner surface of the cell wall facing to the lumen is highlighted in red. The indicated black scale bar is 10 µm.
